# Inhibitory effect of concomitantly administered rabies immunoglobulins on the immunogenicity of commercial and candidate human rabies vaccines in hamsters

**DOI:** 10.1038/s41598-022-10281-1

**Published:** 2022-04-21

**Authors:** Marie-Clotilde Bernard, Florence Boudet, Andrea-Clemencia Pineda-Peña, Françoise Guinet-Morlot

**Affiliations:** Research & Development, Sanofi, Campus Mérieux, 1541 Avenue Marcel Mérieux, 69280 Marcy L’Etoile, France

**Keywords:** Immunology, Vaccines

## Abstract

The World Health Organization protocol for rabies post-exposure prophylaxis (PEP) recommends extensive wound washing, immediate vaccination, and administration of rabies immunoglobulin (RIG) in severe category III exposures. Some studies have shown that RIG can interfere with rabies vaccine immunogenicity to some extent. We investigated the interference of RIG on a next generation highly purified Vero cell rabies vaccine candidate (PVRV-NG) versus standard-of-care vaccines in a previously described hamster model. The interference of either human (h) or equine (e) RIG on the immune response elicited by PVRV-NG, Verorab^®^ (purified Vero cell rabies vaccine, PVRV), and Imovax^®^ Rabies (human diploid cell rabies vaccine; HDCV) was evaluated using the 4-dose Essen PEP regimen. The anti-rabies seroneutralizing titers and specific serum IgM titers were measured by fluorescent antibody virus neutralization test and enzyme-linked immunosorbent assay, respectively, for the vaccines administered with or without RIG. The RIG interference on PVRV-NG, observed transiently at Day 7, was similar to that on PVRV and tended to be lower than that on HDCV using both read-outs. In summary, the results generated in the hamster model showed that RIG induced similar or less interference on PVRV-NG than the standard-of-care vaccines.

## Introduction

Rabies is a major neglected tropical disease present across continents and predominantly affects vulnerable rural populations in Asia and Africa^1,2^. Rabies is an acute progressive viral encephalomyelitis, estimated to cause 59,000 human deaths and over 3.7 million disability-adjusted life years (DALYs) loss every year^2,3^. Dogs are the main source of human rabies, contributing to 99% of all rabies transmissions to humans^3^. There is no cure for rabies; hence, timely and proper post-exposure prophylaxis (PEP) is needed to effectively prevent clinical manifestations of rabies. Standard PEP consists of chemical or biological inactivation of the virus at the site of exposure, followed by a series of vaccine injections^2^. Every year, more than 29 million people worldwide receive PEP for management of rabies exposure, resulting in a notable reduction in mortality rate^3^. Passive immunization with human (h) or equine (e) rabies immunoglobulin (RIG) at the site of the wound in the PEP regimen is considered mandatory for “Category III” exposures corresponding to severe wounds^2^. RIG is injected directly to neutralize the virus before an active response of the vaccine is augmented in individuals who have not previously been vaccinated against rabies^2^. eRIG is preferentially used in endemic countries compared with hRIG due to its low cost; both have shown similar clinical outcomes in preventing rabies^2^. The maximum RIG dose is 20 international units (IU)/kg and 40 IU/kg of body weight for hRIG and eRIG, respectively^2^. The dose should not exceed the recommended limit as it may interfere with the active antibody responses. The RIG should be administered as soon as possible after the rabies exposure in the wound up to the seventh day post-exposure when active antibody response to rabies vaccine has started to develop.

Vaccination is the only effective preventive measure against rabies and rabies vaccines can be administered either as pre-exposure prophylaxis or PEP using different regimens or schedules^2^. The World Health Organization (WHO) recommendations for intra-muscular (IM) PEP include mainly the 4-dose Essen regimen in individuals not previously immunized (1 IM injection at Days 0, 3, 7, and between Days 14 and 28)^2^, although the 5-dose Essen regimen with a fifth dose at Day 28 may still be recommended in some national guidelines. Other IM and intradermal (ID) schedules are also recommended^2^. Several rabies vaccines have been developed, including but not limited to human diploid cell rabies vaccine (HDCV, Imovax^®^ Rabies, Sanofi) in the 1970’s and purified Vero cell rabies vaccine (PVRV, Verorab^®^, Sanofi) in the early 1980’s. Increased disease awareness and development of next generation vaccines are warranted to effectively prevent the infection. With the aim to continue the legacy of innovation, Sanofi is developing a serum-free, highly purified Vero cell rabies vaccine (PVRV-NG) to provide a next generation (NG) of rabies vaccine using the state-of-the-art technology^4,5^. The PVRV-NG vaccine has been developed using the same Pitman-Moore viral strain as PVRV and HDCV^4^. It is a highly purified form of PVRV, without human or animal-derived components (in particular, no human or animal-derived serum or human serum albumin) and antibiotic-free^4^. In addition, the process involves the reduction of residual cell substrate deoxyribonucleic acid (DNA) to ≤ 100 pg/dose. PVRV-NG is currently under Phase III clinical development.

Commercial rabies vaccines are among the most immunogenic vaccines, rapidly inducing high rabies virus neutralizing antibody (RVNA) levels in response to the rabies virus G protein^6^. RVNA titers of ≥ 0.5 IU/mL as an adequate response to vaccination has been accepted by the WHO and regulatory agencies as study endpoints in clinical trials of novel rabies vaccines^2,7^. In most individuals, this level is reached within a few weeks of the first vaccine dose^2^. In PEP regimen with co-administration of RIG, the delay in immune response development to rabies vaccine may be increased due to transient immuno-suppressive effect of the RIG regardless of the vaccination regimen used. Results from clinical trials have shown seroconversion ranging between 80 and 100% at Day 14, whereas in real-world observational cohort studies the seroconversion ranged between 93 and 100% even at Day 28 using IM PEP regimens with different licensed vaccines co-administered with RIG^8–16^. These data indicate that, in clinical trials and observational studies, seroconversion of 100% was not always achieved at Day 14 when RIG was co-administered with the vaccine.

Therefore, in the context of developing a new rabies vaccine, the objective of the study was to evaluate the immunogenic interference induced by RIG on the PVRV-NG vaccine candidate versus that seen with PVRV and HDCV, the two commercially available rabies vaccines considered as standard-of-care, in a previously described hamster model using PEP 4-dose Essen regimen.

## Materials and methods

### Animals

All experiments were performed on female Syrian golden hamsters in animal facilities accredited by the Association for Assessment and Accreditation of Laboratory Animal Care (AAALAC) International. The preliminary experiment and experiment 1 were performed at Voxcan, France and experiment 2 was conducted at Sanofi, Marcy L’Etoile, France. For preliminary experiment and experiment 1, the project and the study protocols were approved by the Ethics Committee of Voxcan France. For experiment 2, the experimental setup was reviewed and preapproved by the Ethics Committee #11 of Sanofi. All experiments were conducted in accordance with the European Directive 2010/63/EU. This study is reported in accordance with the Animal Research: Reporting of In Vivo Experiments (ARRIVE) guidelines.

Female Syrian golden hamsters were purchased from Janvier Labs (France). They were housed in clean polypropylene cages (3 hamsters per cage) and fed standard irradiated laboratory food and water ad libitum. Hamsters were acclimated to their designated housing for 1 week before vaccination. Environmental enrichment was provided by addition of wood brick, paper strip, and cardboard tube for the preliminary experiment and experiment 1, and one tube cardboard, a wheel of exercise, and a square of nestlet type in each cage for experiment 2. At the time of experiments, hamsters weighed 100 g average at Day 0 of the experiment and were around 8 weeks of age. Animals were randomly assigned to treatment or control groups by cage. Additionally, randomization of the allocation of cages was performed according to an internal statistical software. Allocation of 12 animals per treatment group was defined according to a study by De Benedictis et al.^17^. The decision to allocate 6 (experiment 1) or 3 (experiment 2) animals to each of the RIG control groups were based on the findings of the preliminary experiment and corresponding ethical reasons. No blinding of investigator was implemented. No samples nor animals were excluded from the analyses.

The animals were administered with vaccines and immunoglobulins and adverse events were not expected. Animals were observed daily for clinical symptoms with attention to the injected hind legs. Even though serious clinical symptoms were not expected, several ethical endpoints were defined prior starting the study in compliance with the ethical committee recommendations to avoid animal suffering (body weight loss > 20% of the maximal body weight, degradation of the general status, high lesion at the injection site) would have immediately led to euthanasia if one of the endpoints was reached.

### Vaccines and rabies immunoglobulins

All vaccines used in this study were human inactivated whole virus vaccines from Sanofi (Lyon, France). HDCV is produced in human diploid cells (i.e., Medical Research Council cell strain 5 [MRC-5] cells), and PVRV is produced in Vero cells. The potency of all the vaccines used was of ≥ 2.5 IU/dose, determined by National Institute of Health (NIH) mouse potency tests. Each of the vaccines consisted of a freeze-dried formulation presented in a single-dose vial. The powder was reconstituted with 1 mL diluent for HDCV and 0.5 mL diluent for each of PVRV and PVRV-NG. To compare the 3 vaccines using the same vaccine dosage and considering that the largest volume possibly injectable by IM route in hamsters was of 100 µL, we choose to administer 1:10 human dose (HD) for all the vaccines. The hRIG and eRIG were from Sanofi (IMOGAM^®^ Rabies-HT, Lyon, France) and The Thai Red Cross Society (TRCS ERIG^®^, Bangkok, Thailand), respectively.

### Experiments

**Figure 1:**
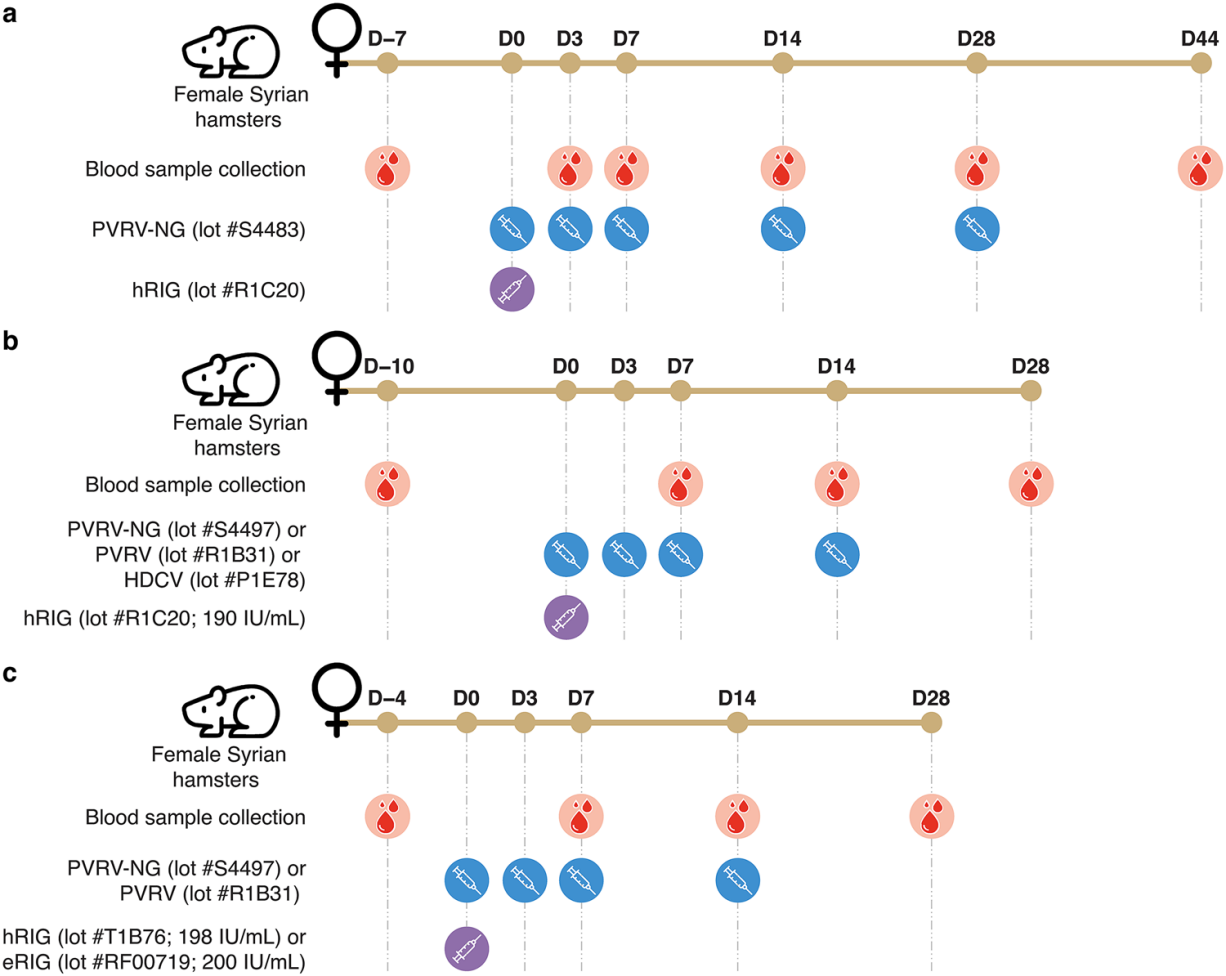
Schematic illustration of vaccination schedule. Female Syrian golden hamsters were injected with standard-of-care rabies vaccines/vaccine candidate (left limb), rabies immunoglobulin (RIG) (right limb), or vaccine + RIG. Blood samples were collected for analysis as illustrated. The preliminary experiment and experiment 1 were performed at Voxcan, France and experiment 2 was conducted at Sanofi, Marcy L’Etoile, France. (**a**) In the preliminary experiment, hamsters were randomized to receive PVRV-NG (n = 12), human RIG (hRIG; n = 12), or concomitant injections of PVRV-NG and hRIG (n = 12). Animals were injected IM with a volume of 100 µL (1:5 HD) of PVRV-NG on Days 0, 3, 7, 14, and 28 (as the 5-dose Essen regimen) and/or a volume of 50 µL of hRIG (20 IU/kg) on Day 0. (**b**) In experiment 1, hamsters were randomized to receive PVRV-NG (n = 12), PVRV (n = 12), HDCV (n = 12), hRIG (n = 6), or concomitant injections of any of the vaccines and hRIG (n = 12 in each group). Animals were injected IM with a volume (1:10 HD) of 100 µL of HDCV, or 50 µL of PVRV or PVRV-NG on Days 0, 3, 7, and 14 (as the 4-dose Essen regimen) and/or a volume of 50 µL of hRIG (20 IU/kg) on Day 0. (**c**) In experiment 2, hamsters were randomized to receive PVRV-NG (n = 12), PVRV (n = 12), hRIG (n = 3), equine RIG (eRIG; n = 3) or concomitant injections of any of the vaccines and hRIG/eRIG (n = 12 in each group). Animals were injected IM with a volume (1:10 HD) of 50 µL of PVRV or PVRV-NG on Days 0, 3, 7, and 14 (as the 4-dose Essen regimen) and/or with a volume of 50 µL of hRIG (20 IU/kg) or eRIG (40 IU/kg) on Day 0.

In a previous study, a hamster model was set up to compare monoclonal antibodies for rabies PEP with the conventional hRIG^17^. We conducted a preliminary experiment to adapt this animal model for the evaluation of hRIG interference on the immunogenicity of PVRV-NG with a high dose of the vaccine (1:5 HD). The hamsters were randomized into 3 groups and administered with PVRV-NG, hRIG, or concomitant injections of PVRV-NG and hRIG (Fig. 1a). The animals were injected IM with a volume of 100 µL (1:5 HD) of PVRV-NG in the left quadriceps on Days 0, 3, 7, 14, and 28 (as a 5-dose Essen regimen) and/or with a volume of 50 µL of hRIG (20 IU/kg) in the right quadriceps on Day 0.

Two successive experiments were then performed to compare the suppressive effect of RIG on the immunogenicity of PVRV-NG and the standard-of-care vaccines. In these experiments, vaccines were administered following the 4-dose Essen regimen omitting the fifth vaccine immunization usually performed on Day 28. The results from the preliminary experiment had shown evidence that this late immunization had no influence in the hamster model. In experiment 1, hamsters were randomized into 7 groups and administered with PVRV-NG, PVRV, HDCV, hRIG, or concomitant injection of any of the vaccines and hRIG (20 IU/kg) (Fig. 1b). The animals were injected IM with a volume (1:10 HD) of 100 µL of HDCV, or 50 µL of PVRV or PVRV-NG in the left quadriceps on Days 0, 3, 7, and 14. hRIG was administered similarly as in the preliminary experiment. The experiment 2 was performed using the same design as that of the first experiment to confirm the interference conferred by co-injection of hRIG (20 IU/kg) with PVRV-NG or PVRV vaccines and to assess the interference conferred by co-injection of eRIG (40 IU/kg) with these vaccines (Fig. 1c). The eRIG interference on HDCV was not tested in this experiment as it is not aligned with the most current medical practices. In experiment 2, hamsters were randomized into 8 groups and administered with PVRV-NG, PVRV, hRIG, eRIG, or concomitant injection of any of the vaccines and hRIG/eRIG. For eRIG alone or vaccine + eRIG groups, animals were injected IM with a volume of 50 µL of eRIG (40 IU/kg) in the right quadriceps on Day 0. The other groups were treated similarly as in experiment 1.

Animals were anesthetized with 5% isoflurane for intermediate blood samplings and IM injections. Intermediate blood samples were taken from the retro-orbital sinus. Terminal blood samples (1 mL) were taken by carotid section after chemical anesthesia with a mix of Imalgène (1.36 mg of ketamine) and Rompun (0.9 mg of xylazine) administered under a volume of 300 µL/100 g by intraperitoneal route. Blood samples were incubated overnight at 4 °C or for 1 h at 37 °C, and sera were collected by centrifugation and stored at − 20 °C until analysis.

### Serological responses

#### Neutralizing antibody responses

The neutralizing antibodies in hamster serum samples were measured using the fluorescent antibody virus neutralization (FAVN) test, an adaptation of the rapid fluorescent focus inhibition test (RFFIT)^18^. Both tests are known to be equivalent when performed under good laboratory practices (GLP) and are recognized as the most accurate techniques to quantify RVNA. FAVN was performed according to the World Organization for Animal Health—Office International des Epizooties (OIE) and the Inovalys laboratory (Nancy, France) using threefold dilutions ranging from 1:6 to 1:354,294 of test sera or standard human rabies anti-immunoglobulin reference (RAI) serum (National Institute for Biological Standards and Control). Briefly, 50 µL of samples were mixed with 50 µL of challenge rabies virus (CVS-11) suspension containing 100 cell culture infectious dose 50% (CCID_50_) of rabies virus in microtiter plates and incubated for 60 min in a humified cell culture incubator. After incubation, Baby Hamster Kidney cells (BHK-21; American Type Culture Collection, Manassas, VA, USA; 50 µL, 4 × 10^6^ cells/mL) were added to the virus/serum mixture and incubated for 48 h in 5% CO_2_ at 37 °C. After incubation, cells were washed, fixed with 80% acetone for 30 min at room temperature (RT), and incubated with 50 µL of an appropriate dilution of fluorescein isothiocyanate (FITC)-conjugated anti-rabies antibody (Fujirebio Diagnostics, USA) for 30 min at RT. The content was discarded; the microplates were washed and air-dried at RT prior to reading under a fluorescent microscope. The titer was determined by comparing the results obtained for the hamster sera with those obtained for the anti-RIG standard and were defined as the serum dilution that reduced the number of fields containing infected cells by 50%, calculated using the CombiStats software (Council of Europe, Strasbourg, France). Titers were converted to IU/mL using the RAI as standard reference curve. The starting dilution was 1:6 so that the lower level of detection was of 0.26 IU/mL. All samples with titers below the lower limit of detection (LOD) were assigned a titer of 0.13 IU/mL equal to half of the LOD.

The window of quantification at this 1:6 dilution was comprised between 0.26 and 3881 IU/mL.

#### Enzyme-linked immunosorbent assay (ELISA) response

The induction of rabies-specific immunoglobulin M (IgM) antibodies was assessed by an in-house ELISA using a commercially available anti-hamster µ chain-specific conjugate (i.e. only detecting the IgM class-specific heavy chain portion in the Fc region) and PVRV-NG as the coating antigen^19^. No anti-hamster γ chain-specific conjugate (i.e. only detecting the IgG class-specific heavy chain portion in the Fc region) was commercially available. As an alternative, an anti-hamster total IgG (H + L) conjugate was used which detected both heavy (H) and light (L) chains of IgG antibodies with significant cross-reactivity with IgM antibodies. In summary, the ELISA of specific antibodies in the serum samples was performed with the following protocol. Greiner 96-well microplates were coated overnight at 4 °C with PVRV-NG vaccine in 0.05 M carbonate-bicarbonate buffer pH 9.6. Plates were then blocked for 1 h at 37 °C with 150 µL of phosphate buffered saline (PBS; pH 7.1)-0.05% Tween 20–1% (w/v) powdered skim milk (PBS-Tween-milk). All further incubations were carried out in a final volume of 100 µL, followed by 3 to 4 washings with PBS, pH 7.1–0.05% Tween. Serial two-fold dilutions of the serum samples performed in PBS-Tween-milk, starting from 1/100, were added to the wells and incubated for 90 min at 37 °C. After washings, an anti-hamster IgM µ chain-specific peroxidase conjugate (Abcepta, San Diego, US) or an anti-hamster total IgG (H + L) conjugate (Jackson Laboratories, Baltimore, US) diluted in PBS Tween–1% milk at 1/5000 or 1/2000, respectively, was added and the plates were incubated for another 90 min at 37 °C. The plates were further washed and incubated in the dark for 30 min at RT with a ready-to-use 3,3′,5,5′-tetramethylbenzidine (TMB) substrate. The reactions were stopped with 100 µL of 1 N HCl. The optical density (OD) was measured at 450 to 650 nm with an automatic plate reader (Molecular Devices, San Jose, CA, USA). The blanks (mean value) were subtracted from the data. The antibody titers were calculated using the Excel or SoftMax Pro software and defined as the reciprocal dilution corresponding to an OD value of 1.0. All final titers were expressed in log scale. For each serum sample displaying an OD value below 1.0 at the first tested dilution of 1/100, the titer was defined as the OD value ×100.

### Statistical analysis

Titers induced by vaccine immunization were statistically compared using a mixed model with product (PVRV-NG, PVRV, or HDCV alone or co-injected with hRIG or eRIG) and time as fixed factors and their interactions.

For group comparison (rabies vaccine + RIG versus rabies vaccine alone), a Tukey’s adjustment for multiple testing was performed. Only significant titer differences (i.e., titer decrease ≥ twofold) were considered biologically relevant considering the precision of the techniques.

A margin of error of 5% was used for effects of the main factors and of 10% for the interaction. The residuals of the model were studied to test the validity (normality, extreme individuals, etc.) of the model.

No replacement of missing or invalid values was made, and no imputation was performed for any of the analyses. A p-value < 0.05 was considered to indicate a statistically significant difference.

## Results

### Preliminary experiment to set up the interference hamster model with hRIG and PVRV-NG

**Figure 2:**
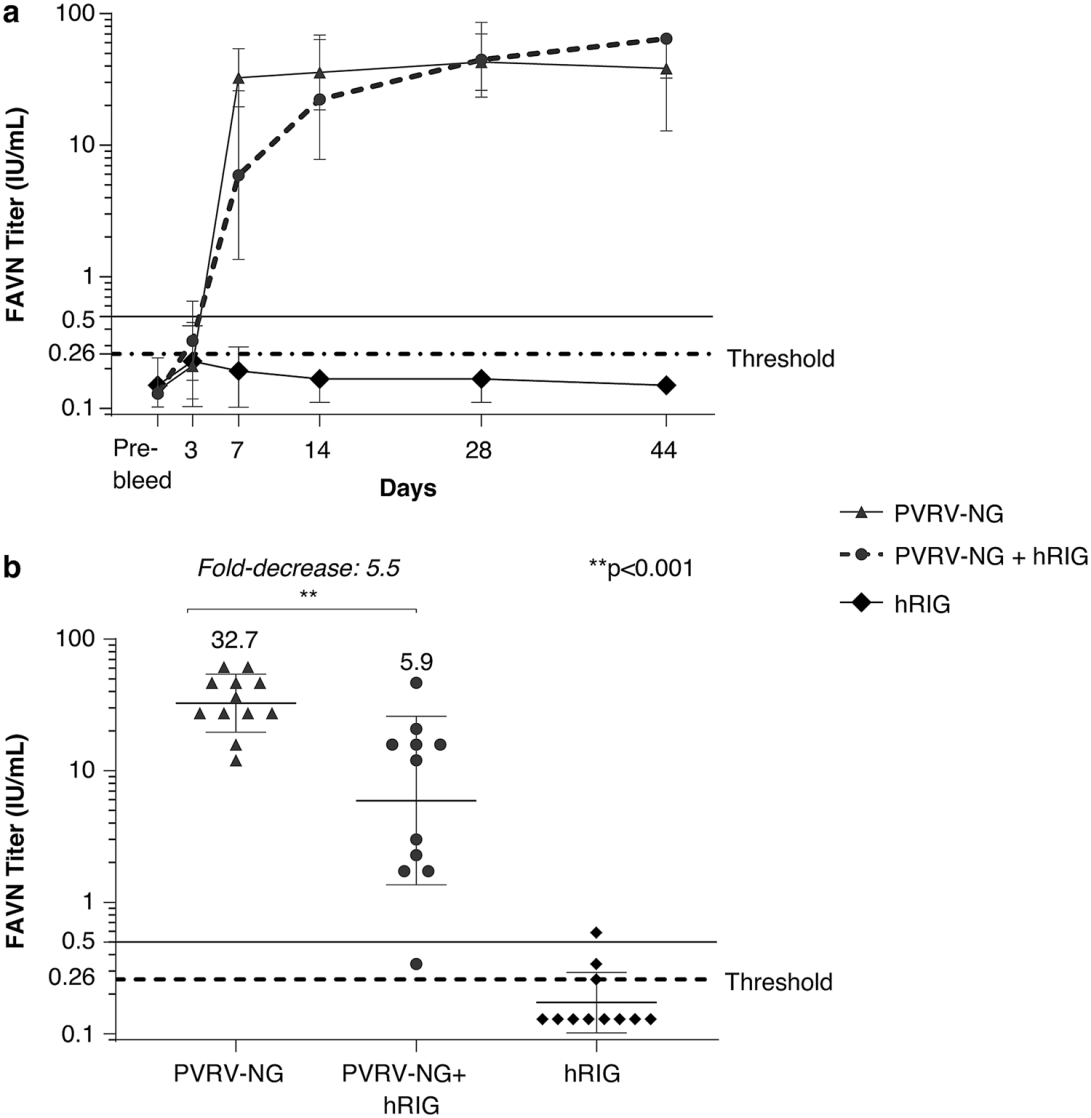
Immunogenicity of PVRV-NG vaccine in presence of human rabies immunoglobulin (hRIG) administered by intramuscular (IM) injection according to post-exposure prophylaxis regimen. In the preliminary experiment to set up the animal model**,** adult female hamsters (n = 12 per group) were vaccinated on Days 0, 3, 7, 14, and 28 by IM injection with 1:5 Human Dose of PVRV-NG in one group, and co-injected with hRIG (20 IU/kg) on Day 0 in another group. (**a**) Serum rabies virus neutralizing antibody (RVNA) levels were monitored by performing a fluorescent antibody virus neutralization test (FAVN) on blood samples collected on Days -7 (7 days prior to Day 0; pre-bleed), 3, 7, 14, 28, and 44. The lower level of detection in FAVN was of 0.26 IU/mL as shown by the dotted line. The continuous black line indicating 0.5 IU/mL is the WHO specified standard serum RVNA titer considered as an adequate immune response to rabies vaccination in humans. Data are expressed as the mean ± SEM. (**b**) Serum RVNA titers measured 7 days after the first immunization.

The results from the preliminary experiment in hamsters showed a statistically significant interference of hRIG with the rabies seroneutralizing response induced by the PVRV-NG vaccine (1:5 HD). The mean FAVN titer measured at Day 7 post-PVRV-NG injections was 5.5-fold lower with concomitant injection of hRIG at Day 0 than without hRIG injection (p < 0.001). The effect of coadministration of hRIG on immunogenicity of PVRV-NG decreased with time and was not significant at later time-points (Fig. 2).

### hRIG interference with PVRV-NG, PVRV, and HDCV vaccines in the hamster model

**Figure 3:**
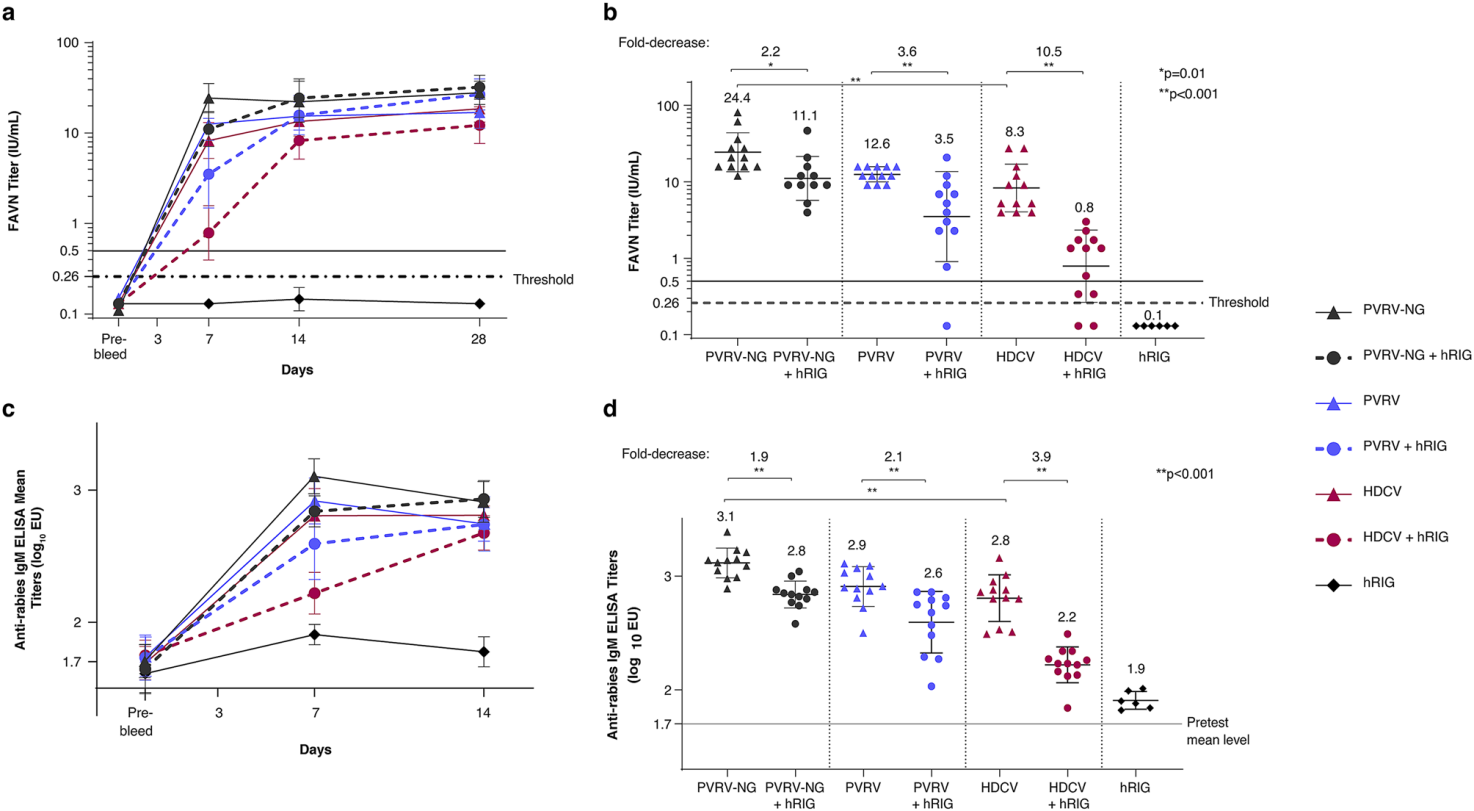
Immunogenicity of PVRV-NG and standard-of-care vaccines in presence of human rabies immunoglobulin (hRIG) administered by intramuscular (IM) injection according to post-exposure prophylaxis regimen. In experiment 1**,** adult female hamsters (n = 12 per group) were vaccinated on Days 0, 3, 7 and 14 by IM injection with 1:10 human dose of PVRV-NG, PVRV, or HDCV and co-injected or not on Day 0 with hRIG (20 IU/kg). (**a**) Serum rabies virus neutralizing antibody (RVNA) levels were measured by fluorescent antibody virus neutralization test (FAVN) on blood samples collected on Days -10 (10 days prior to Day 0; pre-bleed), 7, 14, and 28. The lower level of detection in FAVN was of 0.26 IU/mL as shown by the dotted line**.** The continuous black line indicating 0.5 IU/mL is the WHO specified standard serum RVNA titer considered as an adequate immune response to rabies vaccination in humans. Data are expressed as the mean ± SEM. (**b**) Serum RVNA titers measured 7 days after the first immunization. (**c**) Specific anti-rabies IgM titers were measured by ELISA on blood samples collected similarly as that of FAVN. (**d**) Individual IgM ELISA titers measured 7 days after the first immunization. The mean value obtained with pre-test hamster sera is indicated with a specific continuous line.

The results from experiment 1 showed that neutralizing antibodies achieved high levels as soon as Day 7 for the three vaccines administered alone (PVRV-NG, PVRV, and HDCV) with mean FAVN titers induced by PVRV-NG being significantly higher than that induced by HDCV (p < 0.001) and were not different from those elicited by PVRV (Fig. 3a).

At Day 7, the mean neutralizing titers measured for each of the vaccines with concomitant injection of hRIG were significantly lower than those measured with the corresponding vaccine alone (p = 0.01 for PVRV-NG and p < 0.001 for each of PVRV and HDCV, respectively) with 2.2-, 3.6-, and 10.5-fold-decrease for PVRV-NG, PVRV, and HDCV, respectively, versus vaccine alone. At the following time-points, no more difference in the seroneutralizing titers was evidenced between vaccine groups with or without co-injection of hRIG (Fig. 3b). The group injected with hRIG alone did not show any detectable response at the tested time-points (D7, D14 and 28), as expected from the preliminary study.

The specific rabies ELISA IgM responses were in line with those measured by FAVN titration with mean responses following analogous kinetics as that of FAVN (Fig. 3c). At Day 7, the mean ELISA titers were significantly higher by twofold for PVRV-NG as compared with HDCV (p < 0.001) alone. The mean ELISA titers measured for the groups injected with vaccine and hRIG were significantly lower than those for the corresponding vaccine alone groups (p < 0.001), with 1.9- (below but very close to the twofold limit), 2.1-, and 3.9-fold decrease for PVRV-NG, PVRV, and HDCV vaccines, respectively (Fig. 3d).

### hRIG and eRIG interference with PVRV-NG and PVRV vaccines in the hamster model

**Figure 4:**
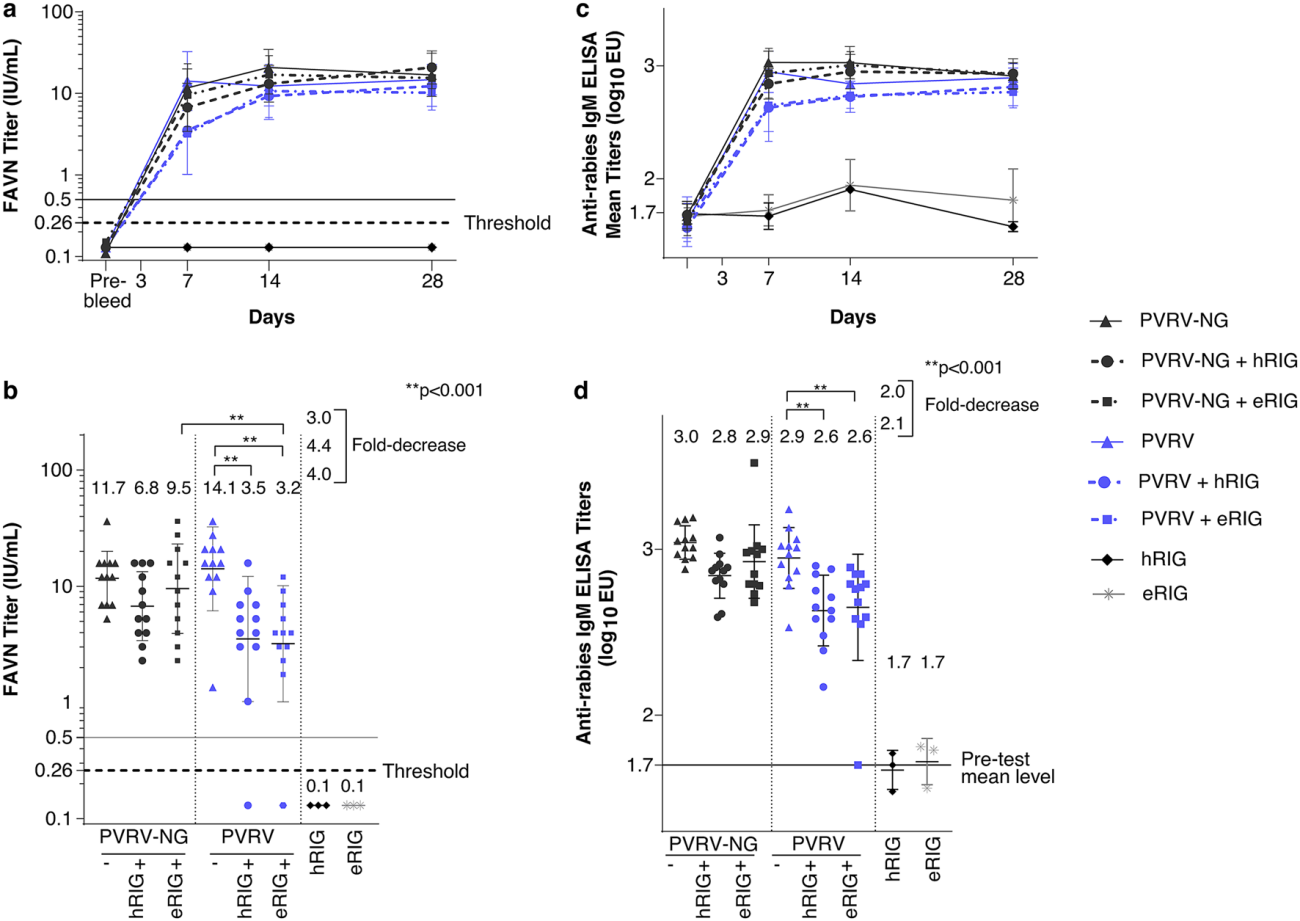
Immunogenicity of PVRV-NG and standard-of-care vaccine in presence of human rabies immunoglobulin (hRIG) or equine rabies immunoglobulin (eRIG) administered by intramuscular (IM) injection according to post-exposure prophylaxis regimen. In experiment 2**,** adult female hamsters (n = 12 per group) were vaccinated on Days 0, 3, 7, and 14 by IM injection with 1:10 human dose of PVRV-NG or PVRV and co-injected or not on Day 0 with hRIG (20 IU/kg) or eRIG (40 IU/kg). (**a**) Serum rabies virus neutralizing antibody (RVNA) levels were measured by fluorescent antibody virus neutralization test (FAVN) on blood samples collected on Days -4 (4 days prior to Day 0; pre-bleed), 7, 14, and 28. The lower level of detection in FAVN was of 0.26 IU/mL as shown by the dotted line. The continuous black line indicating 0.5 IU/mL is the WHO specified standard serum RVNA titer considered as an adequate immune response to rabies vaccination in humans. Data are expressed as the mean ± SEM. (**b**) Serum RVNA titers measured 7 days after the first immunization. (**c**) Specific anti-rabies IgM titers were measured by ELISA on blood samples collected similarly as that of FAVN, D28 was not tested. (**d**) Individual ELISA titers measured 7 days after the first immunization. The mean value obtained with pre-test hamster sera is indicated with a specific continuous line.

In experiment 2, a similar level of interference of hRIG and eRIG on the rabies neutralizing antibody response induced by the PVRV vaccine (4.0- and 4.4-fold decrease on the mean neutralizing titers in the presence of hRIG and eRIG, respectively, p < 0.001) was observed at an early time-point post vaccination (Day 7). As previously observed in experiment 1, such significant decreases were not observed at timepoints post Day 7. Conversely, only a slight decrease of 1.7-fold in the rabies seroneutralizing titer was observed when hRIG were co-injected with PVRV-NG versus PVRV-NG alone but was not deemed significant (i.e., below the twofold decrease limit). In the present experiment, no interference of RIG (either human or equine) on the immunogenicity of PVRV-NG was demonstrated (Fig. 4a,b). Additionally, at Day 7, the mean FAVN titer was significantly higher by threefold for PVRV-NG as compared with PVRV (p < 0.001) in the presence of eRIG.

Estimation of the rabies-specific IgM antibody responses in the vaccinated animals showed a similar trend as that of FAVN, with titer decrease slightly less pronounced by ELISA than by seroneutralization. At Day 7, the geometric mean IgM titers measured for the groups injected with PVRV and hRIG/eRIG were significantly lower than those measured for the groups administered with vaccine alone (p < 0.001), with a titer decrease of 2.1- and 2.0-fold in the presence of hRIG and eRIG, respectively. At the following time-points, no more difference was evidenced between the PVRV groups irrespective of the co-injection or not of hRIG or eRIG (Fig. 4c,d). No significant difference was evidenced between the PVRV-NG groups irrespective of the co-injection of hRIG or eRIG at any of the time points. The groups injected with hRIG or eRIG alone showed very low responses, slightly above the positive threshold.

## Discussion

The delay in immune response development to rabies vaccine by RIG is a well-known phenomenon, which has been described in animal models as well as in humans^20,21^. In clinical trials with various rabies vaccines and different PEP regimens, concomitant injection of RIG at Day 0 was described as inducing a transient interference on the seroneutralizing response measured in the treated subjects^8^. The aim of this study was to compare the interference of RIG on the candidate vaccine versus commercially available rabies vaccines. The results demonstrated that RIG inference with PVRG-NG was similar or even less evident than that measured with our commercial rabies vaccines using a preclinical hamster model that has been described previously^17^.

The aim of the preliminary experiment was to set-up the interference hamster model with a high dose of PVRV-NG (1:5 HD) and hRIG using a 5-dose Essen regimen, the regimen previously recommended by the WHO^7^. A 5.5-fold decrease in the mean seroneutralizing titer in the group administered with both PVRV-NG and hRIG was observed as compared with PVRV-NG alone at Day 7. This clearly indicated the interference of hRIG on the immunogenicity of PVRV-NG. In experiments 1 and 2, we used the currently WHO^2^ 4-dose PEP Essen regimen and a vaccine dose of 1:10 HD, corresponding to the highest vaccine dose in relation to the maximum injectable volume in hamsters. Additionally, it was evident from the preliminary experiment with a 5-dose schedule that the fifth injection had no added value in the hamster model, as previously shown in humans^22^.

To maximize the effect of RIG interference, we decided to inject hamsters with the same dosage of RIG as that used in humans, namely 20 IU/kg for hRIG and 40 IU/kg for eRIG.

The main goal of the study was to infer whether such interference was either lower or similar to that observed on commercial vaccines. The results from experiment 1 showed that PVRV-NG induced statistically significantly higher neutralizing antibodies than that induced by HDCV. Interestingly, the hRIG interference was statistically significant for PVRV-NG, PVRV, and HDCV in this model at Day 7, with 2.2-, 3.6-, and 10.5-fold decrease in mean FAVN titers versus each vaccine alone, respectively. Such an interference decreased with time and was not significant anymore at the later time-points. Whether the RIG was cleared after Day 7 is not known in this experiment. In humans, it has been shown that the peak of hRIG happens at around Day 7 and is gradually decreasing up to around 3 weeks, while the hRIG-induced interference on vaccines can still be seen on Day 14 and is undetectable by Day 42^23,24^.

As the affordability and availability of the hRIG are key issues in endemic countries, eRIG has been used for the PEP regimens in some countries. Thus, experiment 2 was performed to assess the effect of eRIG on the rabies vaccines in the established hamster model using the Essen regimen. Of note, similar interferences were observed on PVRV-NG with eRIG or hRIG, although not statistically significant. However, we noted a statistically significant similar level of interference of hRIG and eRIG on the rabies neutralizing antibody response induced by PVRV with 4.0-fold and 4.4-fold decreases, respectively, in the mean neutralizing titers observed at an early time-point post vaccination (Day 7). Such a titer decrease was not statistically significant anymore at the following time-points. It is to be noted that, in both experiments, consistent levels of interference were highlighted when PVRV was co-injected with hRIG (3.6- and 4.0-fold decrease in experiment 1 and experiment 2, respectively) compared with the vaccine alone. It has previously been reported in a guinea-pig model that when rabies-specific antisera were concomitantly administered with rabies vaccine, the ability of animals to mount a protective immune response against the vaccine was lower for homologous than for heterologous antisera (i.e. raised in the same or in a different animal species, respectively)^21^. The stronger interference of passively transferred homologous antibodies on the vaccine response was associated with their longer persistence in the circulation than that of their heterologous counterparts. The high interference effect of homologous rabies-specific antibodies on rabies vaccine response was also observed with monoclonal antibodies in a mouse model^25^. Future studies will be needed to evaluate such interference on the vaccines used in this study. Noteworthy, both eRIG and hRIG, assessed herein, were heterologous immunoglobulins to the hamster model.

In the preliminary experiment, the seroneutralizing titer elicited at Day 7 by PVRV-NG in the presence of hRIG was 5.5-fold lower than that induced by the vaccine alone. In experiments 1 and 2, the interference of both hRIG and eRIG on PVRV-NG-induced seroneutralizing and ELISA titers was negligible. Indeed, titer fold decrease compared with vaccine alone was close to or below the 2.0-fold threshold that we considered as biologically relevant given the variability of biological responses associated with both techniques and animals. Although the clinical batch of PVRV-NG used in the preliminary experiment was different from that used in the experiments 1 and 2, both batches contained the same amount of G-protein. The 5.5-fold decrease in the mean FAVN titer in the preliminary experiment with 1:5 HD of PVRV-NG vaccine was replicated proportionally in experiment 1 with a 1:10 HD of the vaccine dose and respective 2.2-fold decrease. The decrease in the mean FAVN titer in experiment 2 with 1:10 HD of PVRV-NG vaccine was below but very close to the defined threshold. Above all, irrespective of vaccine interference introduced by concomitant RIG administration, all reported RVNA levels were still maintained well above 0.5 IU/mL at Day 7 onward, which was indicative of an adequate immune response to vaccination. With the data from all the experiments described herein, it can be inferred that hRIG or eRIG induced similar or even less interference on the PVRV-NG vaccine candidate as compared with that evidenced with commercial vaccines. The interference of RIG on PVRV-NG was similar to that on PVRV and tended to be lower than that on HDCV. The reasons of such results are still unknown and warrant further investigation.

The decrease in the antibody titer as measured by ELISA corroborated the findings from the FAVN, with the fold-decrease in titer slightly less pronounced by ELISA than by FAVN seroneutralization. The advantage of the ELISA test is its convenience and rapidity to generate data. Although the seroneutralization test is more laborious and time consuming, it remains the gold standard to measure immunogenicity induced by rabies vaccines.

The induction of rabies-specific IgM antibodies was assessed by an in-house ELISA using a commercially available anti-hamster µ chain-specific conjugate and PVRV-NG as the coating antigen. As no anti-hamster γ chain-specific conjugate was commercially available, anti-rabies IgG antibodies could not be strictly titrated. Alternatively, we used an anti-hamster total IgG (H + L) conjugate capable of detecting IgG and significantly cross-reacting with IgM antibodies. With this conjugate, similar results to those measured with the IgM conjugate were found, indicating that the IgM response was preponderant (data not shown). IgM is indeed the first class of immunoglobulin appearing during onset of an immune response, and such antibodies were of interest to quantify in our hamster model at the tested early time-point (Day 7).

The mechanism explaining the transient interference of RIG on rabies vaccine immunogenicity is still not fully understood. The formation of immunocomplexes between rabies antigens and RIG may temporarily mask vaccine epitopes and/or contribute to antigen opsonization and clearance, thereby lessening immune exposure. Schumacher et al. showed that the pretreatment of mice with a cocktail of murine anti-rabies monoclonal antibodies interfered with the ability of the animals to mount a virus-neutralizing antibody response upon a subsequent rabies vaccination^25^. The authors demonstrated that the duration of this interference increased with the monoclonal antibody concentration and was inversely proportional to the biological half-life of the administered antibodies. As mentioned above, the previously reported stronger interference of rabies-specific homologous antibodies compared to heterologous antibodies^21^ are in line with the shorter biological half-life of the former antibodies over the latter. Interestingly, injection of mice with immunocomplexes of inactivated rabies virus and monoclonal antibodies was shown to negatively impact the activation of rabies virus-specific B cells^25^. It was postulated that the immunocomplexes could exert a negative signaling on immature IgM receptor-bearing primary B cells, preventing them from differentiation into antibody-secreting plasma cells. Whether this mechanism transiently occurred in our present studies is not known.

The results of the present study showed that the potential interference of RIG on PVRV-NG in a hamster model was similar or even less pronounced by our PVRV-NG as compared with our commercial vaccines. In future investigations, the extrapolation of these results needs to be confirmed in human clinical trials to verify the predictivity of the hamster model. The clinical development plan of the vaccine candidate includes a phase III-simulated Essen PEP regimen study in healthy adults that aims: (i) to demonstrate the non-inferiority of PVRV-NG versus PVRV and HDCV vaccines when co-administered with hRIG and (ii) to describe the hRIG interference in an arm of PVRV-NG administered as standalone (NCT03965962). This study will help in further elucidating the extent and nature of RIG interference on the new candidate vaccine especially when the full quantity of hRIG is administered by IM route as the most stringent evaluation in healthy participants. On the contrary, the clinical practice has changed after the WHO 2018 recommendations^2^, in which the RIG is infiltrated into and around the wound and the remaining RIG is no longer administered intramuscularly at a distant site.

## Conclusion

In this study, we confirmed the transient interference of human and equine RIG on the rabies neutralizing antibody responses induced by the PVRV and HDCV commercial vaccines in a hamster model and demonstrated that such interference was similar or even less pronounced for the PVRV-NG vaccine candidate.

## Data Availability

The datasets generated during the current study are available from the corresponding author on reasonable request.
